# Utilisation of drugs for the treatment of psychiatric diseases in the pediatric population: focus on off-label use

**DOI:** 10.3389/fphar.2023.1157135

**Published:** 2023-06-16

**Authors:** Stella Pesiou, Rafel Barcelo, Marc Fradera, Ferran Torres, Caridad Pontes

**Affiliations:** ^1^ European Medicines Agency, Amsterdam, Netherlands; ^2^ Department of Pharmacology, Therapeutics and Toxicology, Universitat Autònoma de Barcelona, Edifici M Campus de la UAB, Bellaterra, Spain; ^3^ Departament of Pediatrics, Gynecology and Obstetrics, and Preventive Medicine, Universitat Autònoma de Barcelona, Edifici M Campus de la UAB, Bellaterra, Spain; ^4^ Unitat Mixta de Neurociència Traslacional I3PT-INc-UAB, Parc Taulí Hospital Universitari, Institut d’Investigació i Innovació Parc Taulí (I3PT-CERCA), Centro de Investigación Biomédica en Red de Salud Mental (CIBERSAM), Sabadell, Spain; ^5^ Department of Medicines, Area of Healthcare Services, Catalan Health Service, Barcelona, Spain; ^6^ Digitalization for the Sustainability of the Healthcare System (DS3), Institut d’Investigacio Biomedica de Bellvitge (IDIBELL), Barcelona, Spain

**Keywords:** children, adolescents, pediatric, psychiatry, drugs, psychotropics, prevalence, off-label

## Abstract

Psychotropics are increasingly used in pediatrics, often as off-label medicines. The guarantees of safety and efficacy are not always granted in clinical practice compared to adult authorised indications. A retrospective observational study was done to estimate the prevalence of psychotropic use in pediatric subjects of Catalonia (Spain). Anonymised data on dispensation of psychotropics to pediatric patients, demography and other related data were obtained by the local healthcare management for the period 2008–2017. Estimation of off-label use was done through description of drug dispensations with no authorised use related to age range. The prevalence of psychotropics was 40.8–64.2 per 1,000 pediatric inhabitants. Hydroxyzine-only represented two-thirds of dispensations, and when removed, the prevalence dropped to 26.4–32.2 per 1,000 pediatric inhabitants. Adolescents and boys were more likely to receive a psychotropic. Psychostimulants had the highest exposure rate, mainly due to methylphenidate. Off-label use was observed in 12% of subjects, corresponding to 4.6% of all dispensed psychotropics with boys being more exposed. The proportion of off-label use vs. labelled use was higher in younger populations. Aripiprazole had the highest off-label frequency. Our data support the frequent reality of off-label use in pediatrics, despite the potential underestimation related to the selected off-label definition. There is an urgent need to systematically ascertain effectiveness and any potential adverse events in the off-label pediatric setting, and to generate valuable information for risk-benefit assessment in these populations where extrapolation from adults is not reliable.

## 1 Introduction

There is a great relevance in ensuring that appropriate efforts are directed to preserve, promote, restore and protect mental health, as a key axis of the overall subject and community wellbeing and functionality (World Health Organization, 2018). Global estimates suggest that roughly 1 in 5 children and adolescents present with a mental health condition, that half of the mental conditions start by the age of 14 years old, while suicide is considered the third leading cause of death in adolescents aged 15–19 years old (World Health Organization, 2021; World Health Organization, 2023). Hence, the epidemiology of mental disorders in the pediatric population still has room for improvement, while few and generally fragmented data are available on the prevalence of the different conditions and their treatments, since many countries do not have the appropriate information systems to obtain them.

Children and adolescents have been traditionally considered a neglected population from a therapeutic perspective. Reasons include the intrinsic clinical difficulty to identify mental signs and symptoms in pediatric patients along with the developmental changes, the lack of awareness on the frequency and importance of mental conditions in children, social stigma on mental disorders (World Health Organization, 2013), as well as several other reasons acting as barriers to the development of new drugs and evidence-based treatment options for this population (Koelch et al., 2008; Hoffman et al., 2014).

Data on the use of drugs are important to understand the epidemiology of a specific health problem, especially in a population for which the participation in clinical studies is often not considered ethical or easily manageable. Therefore, real-world evidence can be useful for prescribers and patients by complementing the information derived from randomised clinical trials. Moreover, data on effectiveness in clinical practice may complement efficacy and safety data in groups of patients not included in clinical trials, wider samples and diverse cultural and social settings, allowing to clarify uncertainties and to complete missing information (Lapeyre-Mestre et al., 2013). In a literature search for studies describing the use of psychotropics in children and adolescents in Spain, we found only one population-based study, but the subjects were of the age of 15 years and above (Barceló et al., 2016).

We aimed to describe the use of psychotropics in the pediatric population in the region of Catalonia (Spain) and their trends in time, in order to identify the main areas of exposure to psychotropic drugs used in this vulnerable population in our setting.

## 2 Materials and methods

We conducted a retrospective observational population-based quantitative study of psychotropic consumption in the population below 18 years of age residing in the region of Catalonia (Spain) and covered by the social security. Pharmacy billing data from the Catalan Health Service (*CatSalut*) for a 10-year period (2008–2017) were used and linked as per the single identification number of the Catalan Health Service to the data contained in the central insurance registry for demographics and insurance status. The pharmacy reimbursement data are considered representative of the region’s population, since all residents in Catalonia (approximately 7.72 million, 16.4% of Spain’s total population) (Instituto Nacional de Estadística, 2018) are covered by the public system. The data used for this analysis were provided by the *Agència de Qualitat i Avaluació Sanitàries de Catalunya* (*AQuAS*) through the *PADRIS* program (*Programa públic d’analítica de dades per a la recerca i la innovació en salut a Catalunya*) upon records linkage, by personnel not linked to the study team and anonymised the data following a procedure aiming to maintain the data confidentiality via double encryption and removal of personal identifiers.

The extracted data were reviewed for their quality and completion. No further action was done concerning the missing data as their collection was not feasible due to the retrospective design of the study. Before the actual analysis, duplicate cases were detected and deleted. The analysis of the study was descriptive: categorical variables were described using frequencies and percentages, while the use of psychotropics was defined as per the pharmacy dispensed number of medicines following their prescription by a physician and given as prevalence by 1,000 Catalan inhabitants aged less than 18 years. As psychotropics we included the following ATC groups as defined by WHO: antiepileptics (N03A), antipsychotics (N05A), anxiolytics (N05B), hypnotics/sedatives (N05C), antidepressants (N06A), psychostimulants (N06B) and drugs used in addictive disorders (N07B). The off-label use was approached through analysis of drugs dispensed out of the authorised age range (if any) for each product as defined from products labelling, in a subset of data between 2015 and 2017 and was presented using percentages. The listing of the most frequently off-label dispensed psychotropics was described for 1 year (2017).

The statistical analysis was performed using the statistical package SAS v9.4 (SAS Institute, Cary, NC, United States).

The protocol of this observational study was authorised by the relevant ethics committee in Catalonia, but no further authorisation or signed patient consents were needed according to national regulation.

## 3 Results

### 3.1 Prevalence of psychotropics use

During the study period (2008–2017) 449,196 subjects with at least one psychotropic drug dispensed from the Catalan health reimbursement system were identified in the concerned region; 137 patients were excluded as they had potential errors as regards the sex variable, and therefore our initial sample was finally of 449,059 pediatric subjects.

The annual prevalence of use of psychotropics was between 40.8 and 64.2 per 1,000 pediatric inhabitants with at least one psychotropic dispensed within the 10-year period of this study. Pediatric subjects under hydroxyzine-only treatment represented two-thirds of the dispensations. After removing the hydroxyzine-only dispensations data, the prevalence of psychotropic use in the pediatric population of this targeted exposure was between 26.4 and 32.2 per 1,000 pediatric inhabitants. Adolescents and boys were more likely to receive a psychotropic drug. Table 1 presents in detail the prevalence of use in the Catalan pediatric population of our setting in total and per age groups.

**TABLE 1:** Prevalence of psychotropics use in Catalonia’s pediatric population from 2008 to 2017 (per 1,000 pediatric inhabitants).

	2008	2009	2010	2011	2012	2013	2014	2015	2016	2017
*Pediatric inhabitants*	***1,281,777***	***1,322,462***	***1,346,516***	***1,367,382***	***1,384,978***	***1,389,763***	***1,386,458***	***1,388,261***	***1,391,507***	***1,398,400***
Any exposure										
*All*	***63.7***	***40.8***	***53.6***	***60.7***	***60.5***	***56.9***	***63.7***	***64.2***	***62.0***	***55.2***
Girls - Boys	57.6–69.5	33.5–47.6	46.5–60.3	52.4–68.4	52.0–68.4	48.7–64.7	55.8–71.1	56.4–71.4	54.5–68.9	47.7–62.1
Target exposures										
*All*	***26.4***	***27.2***	***30.9***	***31.3***	***31.5***	***31.8***	***32.2***	***31.4***	***30.3***	***29.6***
Girls - Boys	19.4–32.9	19.8–34.1	23.3–37.9	22.9–39.2	22.8–39.6	23.1–39.9	23.9–39.9	23.5–38.9	22.6–37.5	22.2–36.6
*< 1* *year*	***0.0***	***0.0***	***1.3***	***1.1***	***1.7***	***2.4***	***2.2***	***2.1***	***1.7***	***1.9***
Girls - Boys	0.0–0.0	0.0–0.0	1.3–1.4	0.9–1.3	1.3–2.1	1.9–2.7	1.8–2.5	1.9–2.3	1.6–1.7	1.6–2.3
*1–2* *years*	***1.0***	***4.4***	***8.3***	***7.0***	***5.3***	***5.0***	***4.7***	***4.8***	***4.9***	***4.6***
Girls - Boys	0.9–1.1	3.9–4.8	7.4–9.1	6.1–7.8	4.7–6.0	4.2–5.9	3.8–5.6	4.0–5.5	4.4–5.3	4.2–4.9
*3–5* *years*	***14.9***	***11.4***	***12.0***	***11.0***	***8.9***	***7.9***	***6.7***	***6.2***	***5.8***	***5.6***
Girls - Boys	12.2–17.5	9.7–12.9	9.7–14.2	9.0–12.8	6.8–10.9	6.0–9.7	5.5–7.9	5.1–7.3	4.6–6.8	4.4–6.8
*6–8* *years*	***21.4***	***18.4***	***23.7***	***22.9***	***21.0***	***23.8***	***22.4***	***19.9***	***17.7***	***16.8***
Girls - Boys	17.2–25.5	13.5–23.1	16.1–30.9	14.8–30.5	13.7–27.9	14.4–32.6	13.8–30.5	12.1–27.2	10.9–24.1	10.3–22.8
*9–11* *years*	***37.0***	***38.9***	***44.4***	***45.5***	***43.7***	***45.4***	***43.2***	***40.5***	***37.5***	***34.8***
Girls - Boys	25.9–47.5	24.8–52.2	28.5–59.4	28.8–61.4	26.9–59.7	27.1–62.6	26.1–59.3	24.2–55.8	22.6–51.4	20.8–47.8
*12–14* *years*	***45.6***	***47.8***	***50.9***	***54.2***	***55.8***	***57.2***	***58.8***	***58.0***	***55.3***	***53.2***
Girls - Boys	30.6–59.8	29.8–64.8	33.3–67.3	34.9–72.0	36.4–73.9	37.9–75.2	39.8–76.8	39.3–75.6	37.0–72.6	35.9–69.5
*15–17* *years*	***48.5***	***55.3***	***60.4***	***62.8***	***69.2***	***63.5***	***66.8***	***66.1***	***65.2***	***63.7***
Girls - Boys	36.8–59.5	46.7–63.3	56.1–64.4	55.6–69.6	60.5–77.2	59.3–67.3	63.5–69.7	62.9–69.0	61.1–69.0	59.5–67.6

Bold italic figures refer to the overall (Girls+Boys) data

**FIGURE 1:**
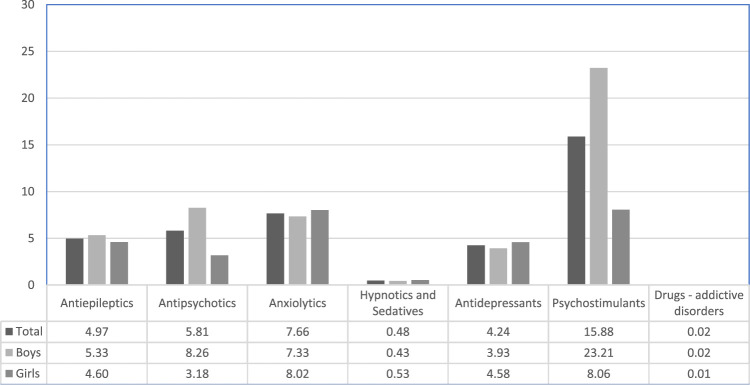
Prevalence (per 1,000 inhabitants) of psychotropics use by ATC groups..

Psychostimulants had the highest prevalent exposure in our setting (Figure 1) and boys were much more exposed compared to girls. Methylphenidate was found to be the most prevalent psychotropic dispensed to the Catalan pediatric population, but antipsychotics and antidepressants were also highly ranked (see Supplementary Material S1).

#### 3.2 Off-label use

The subset of the pediatric population used for the off-label analysis consisted of 66,824 outpatient pediatric subjects with at least one psychotropic drug dispensed between 2015 and 2017, corresponding to 950,395 drug dispensations. Off-label use was observed in 12% of pediatric subjects and was more frequent in boys (Table 2).

**TABLE 2:** Off-label use considering labelling age range and most frequently off-label drugs.

***a. Off-label use considering labelling age (patients)***						
		**Girls**	**Boys**		**Total**	
		**n (%)**	**n (%)**		**n (%)**	
In the authorised age range		21,910 (82.93)	36,880 (91.28)		58,790 (87.98)	
Out of authorised age range		4,510 (17.07)	3,524 (8.72)		8,034 (12.02)	
**Overall**		26,420	40,404		66,824	

***b. Most frequent off-label drugs in 2017***						
**ATC**			**Dispensed psychotropics** (*n = 310,078*)			
**Code**	**Name**		**n**	**%**		**D/P^a^**
N05AX12	aripiprazole		5,496	1.77		4.8
N05AH04	quetiapine		4,380	1.41		3.9
N05AH03	olanzapine		2,654	0.86		3.6
N06AB10	escitalopram		1,165	0.38		3.6
N05BA12	alprazolam		1,036	0.33		1.8
N05AX13	paliperidone		1,024	0.33		6.7
N05BA09	clobazam		872	0.28		6.6
N06AB04	citalopram		743	0.24		3.5
N06AX11	mirtazapine		659	0.21		2.9
N03AF02	oxcarbazepine		622	0.20		6.5

^a^ Dispensed drug per patient ratio.

**FIGURE 2:**
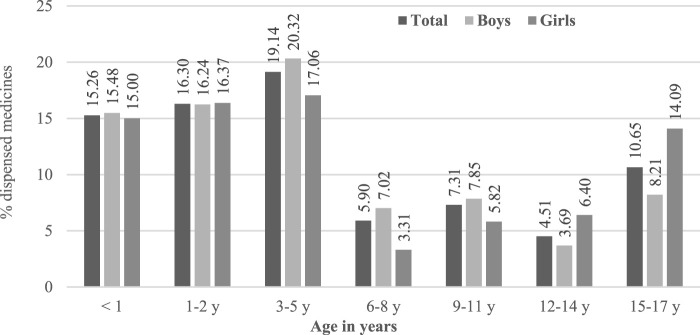
Off-label use considering labelled age-range, by age groups of subjects.

The proportion of off-label use vs. labelled use was higher in younger populations as described in Figure 2. Considering data only in 2017, aripiprazole was the active substance most frequently used under an off-label status followed by two other antipsychotics (Table 2).

## 4 Discussion

### 4.1 Psychotropic use in paediatrics

We analysed anonymised data on dispensed drugs covered by the national health system of Catalonia. The overall prevalence of psychotropic use was between 4.1% and 6.4% in Catalonia and was led by a high rate of short-term use of hydroxyzine, an H1 antihistaminic which is used for several acute indications such as skin allergies and itching. To focus the analysis on psychotropic use in mental conditions, subsequent analyses excluded patients who had only hydroxyzine dispensations. In the population with this targeted exposure, the prevalence of psychotropics use in the pediatric population ranged between 2.6% and 3.2%, and was more frequent in adolescents as well as in boys, mostly due to the use of psychostimulants. A significant amount of the dispensed psychotropic drugs was used out of the authorised age range, with higher proportions of the overall use being not covered by labelling age range in younger patients. Aripiprazole was the most frequent drug dispensed off-label, since in Europe it is authorised for use in children older than 13 years of age.

The rates of exposure to psychotropic drugs in Catalonia were found to be within the ratio reported in other European countries as well as the United States of America (Zito et al., 2003; Safer et al., 2004; Zito et al., 2008; Zoëga et al., 2009). A study in France reported a lower prevalence for the year 2010 compared to Catalonia (25.0 vs. 30.9 per 1,000 respectively) (Kovess Masfety et al., 2015). The current study also demonstrated that boys are more exposed to psychotropics than girls, confirming the trend that has been reported in previous studies (Zito et al., 2003; Zito et al., 2008; Kovess Masfety et al., 2015; Hartz et al., 2016; Piovani et al., 2016). Psychostimulants use rate was 15.88 per 1,000 inhabitants, similar to the rates reported in Germany (7.1–22.0 per 1,000) (Zito et al., 2008; Bachmann et al., 2017), the Netherlands (1.5–39.0 per 1.000) (Schirm et al., 2001; Faber et al., 2005; Zito et al., 2008; Bachmann et al., 2017) and Israel (7.0–25.0 per 1,000) (Vinker et al., 2006), slightly higher than Denmark (0.9–15.0 per 1,000) (Pottegård et al., 2012; Bachmann et al., 2017) and lower than the ones reported in Iceland (21.7–28.4 per 1,000) (Zoëga et al., 2009), whereas in France (2.0 per 1,000) (Kovess Masfety et al., 2015), Italy (0.1–1.9 per 1,000) (Piovani et al., 2016) and the United Kingdom (3.0–5.0 per 1,000) (Bachmann et al., 2017), the reported prevalence rates for psychostimulants were lower.

We had exhaustive information on drug dispensation from the public healthcare in Catalonia for a period of 10 years, which can be considered representative for the region since the system covers almost all inhabitants and was fully available across the whole study period (Modol et al., 2017). Prescription data might be a better option to describe the use of drugs, since they may involve information on indication. Advantages of using pharmacy invoicing data render the observations to be more representative of the actual exposure in specific drugs, considering that these data enclose information on patients that have actually collected the prescribed treatment. Hence, data on reimbursement can define better the prevalence on the use of a drug, even though one cannot still be reassured in absolute terms about the actual adherence to the dispensed medications. While the actual use may differ compared to the purchase of drugs and may also change in time, invoicing data are useful to define the trends and any relevant change throughout the evaluated period. Nonetheless, the extent of information in the present study was limited, since the type of variables available in healthcare databases is predetermined and focused on the management of invoicing and healthcare services (Andersen, 2014). Another limitation from the source of the data used here is the fact that databases of reimbursements are limited to drugs in need of prescription; since in Spain all psychotropics are prescription-only medications, this limitation is unlikely a source of bias in our study. Linking the use of drugs and the disease for which they have been prescribed, would have permitted to analyse more in depth the proportion of off-label use, considering the indication of the treatment. However, since these data were not available for the current study, we approached the off-label analysis through the perspective of the age range only.

### 4.2 Off-label use of psychotropics

Medication used under an off-label status is often considered a common practice in pediatric patients which imposes this vulnerable population into a high-risk situation due to uncertainties in the efficacy and safety of the concerned treatments. Psychotropic medication is often not studied in underaged patients due to a large list of reasons like barriers regarding pharmacological treatments to obtain evidence-based treatment options for children (Koelch et al., 2008) or lower epidemiology of mental health disorders as opposed to adults. The lack of research for many decades has led to a situation where many drugs currently used in children are lacking clinical evidence in pediatric conditions, and physicians need to extrapolate their practice from the adult studies or the knowledge acquired by clinical experience along the years, to be able to treat their pediatric patients (Kern, 2009).

The off-label use in both Spain (and subsequently Catalonia) is somehow regulated, as there are legal measures in place to regulate requirements for an off-label use. A law established in 2009 states that a drug could be given as off-label in both hospitals and primary care settings, provided that physicians justify the need and absence of commercialised alternatives suitable for the patient, as well as they should obtain the consent from the patient after informing him or her on the benefits and risks (Ministerio de SanidadPolítica Social, 2009). In certain circumstances for repeated uses, physician’s obligations may be waived if approved protocols are in place (Ministerio de SanidadPolítica Social, 2009). However, available evidence in Europe demonstrates that there is a relationship between off-label use in children and an increased percentage and severity of adverse drug reactions (ADRs); in particular, neuro-psychiatric ADRs are detected more frequently in children compared to adults (European Medicines Agency, 2004). According to other studies, between 23% and 60% of all ADRs in children may be related to drugs used out of their authorised conditions (Neubert et al., 2004; Cuzzolin et al., 2006; Fabiano et al., 2012).

The off-label use of psychotropics in pediatric population has been previously reported in several countries. The extent of off-label use depends on the setting and the applied off-label definition: small studies used to focus on a given indication or drug, provide more detailed descriptions and include information on formulation, dose and indication that allow a more accurate definition of the off-label use, hence leading to higher rates of off-label use than the ones identified in our study (Braüner et al., 2016; Deng et al., 2018; Kornø and Aagaard, 2018). In the present study, the data did not permit a very precise definition of off-label use, since they did not allow linking dispensed medications to a clinical indication. In addition, information on the age of the subjects was received as being aggregated into fixed age groups due to confidentiality purposes. Therefore, we had to limit the analysis of the off-label use to the age range for which each active substance was approved, leading to a partial description of the off-label rates driven only by the approved age and not by the approved indication of psychotropics.

Considering all these limitations, we observed that 12% of Catalan pediatric subjects received at least one psychotropic as off-label related to any pediatric approval of at least one product with the same active substance. Our study results may be compared only to one study reporting the off-label psychotropic prescriptions in the Icelandic pediatric population (Zoëga et al., 2009). Nonetheless, the much higher off-label use in Iceland compared to the one described in our sample, can be explained by our methods based on age only and the study periods that do not coincide.

To date there is still a huge need of appropriate and consistent regulatory information for the use of available psychotropic drugs in children and adolescents. We have shown that such use of available drugs is far from being rare, but this off-label use is currently done empirically and in the absence of the regulatory guarantees that are granted through strict product labelling processes (Christiansen et al., 2022). The present study provide an overview on the psychotropic use and highlights that pediatric patients are in need of special attention. The data generated in this study could be a basis to start working towards an improved environment for pediatric healthcare with treatments having the guarantees of quality, safety and efficacy for pediatric patients at the same level as the ones existing for adults. Our study could be considered as a starting point to identify the most frequent off-label use in pediatric mental healthcare, in order to inform guidelines on how psychotropic off-label use can be minimised, especially for those substances with no robust evidence in the particularly vulnerable underaged patient group. Moreover, the information described in our study could be also useful to regulatory authorities in order to define if there is still a need to further harmonise the requests for granting or extending an authorisation in psychiatric indications for the pediatric population, and subsequently to foster further the implementation of the pediatric regulation in Europe which was recently under revision (European Medicines Agency, 2021). Real-world evidence data could also play an important part to fill in the gap especially for all those medicines that are used for a long period under an off-label status in this patient population. Finally, our data could support actions aimed to align the incentives for pharmaceutical industry with the needs to extend the labelling of existing psychotropics, many under an off-patent status and approved for use only in adults, and thus to regulate current established uses and patient age groups (Lepola et al., 2020).

## Data Availability

The raw data supporting the conclusions of this article will be made available by the authors, without undue reservation.
