# Supplementary material for: Utilisation of drugs for the treatment of psychiatric diseases in the pediatric population: focus on off-label use
Source: Front Pharmacol. 2023 Jun 16;14:1157135. doi: 10.3389/fphar.2023.1157135 (PMC10312111; doi:10.3389/fphar.2023.1157135)
Supplement: Supplementary file 1 [file DataSheet1.docx]

Supplementary Material

Utilisation of drugs for the treatment of psychiatric diseases in the paediatric population: focus on off-label use

Stella Pesiou, Barcelo Rafel, Marc Fradera, Ferran Torres, Caridad Pontes

*** Correspondence:** Ferran Torres: Ferran.Torres@uab.cat

# Supplementary Data

Heatmap table for the most prevalent medicines used by age strata in Catalonia (Spain) from 2008 to 2017 - target exposures.

|  |  | **Overall** | **Prev. (‰)** | **Girls** | **Prev. (‰)** | **Boys** | **Prev. (‰)** |
| --- | --- | --- | --- | --- | --- | --- | --- |
| **< 1 year** | **1** | N03AG01: Valproic acid | 3.5 | N05BA01: Diazepam | 3.2 | N03AG01: Valproic acid | 4.1 |
|  | **2** | N05BA01: Diazepam | 3.5 | N03AG01: Valproic acid | 2.9 | N05BA01: Diazepam | 3.8 |
|  | **3** | N03AX14: Levetiracetam | 2.8 | N03AX14: Levetiracetam | 2.6 | N03AX14: Levetiracetam | 3 |
|  | **4** | N05BB01: Hydroxyzine | 1.7 | N03AG04: Vigabatrin | 1.2 | N05BB01: Hydroxyzine | 2.1 |
|  | **5** | N03AG04: Vigabatrin | 1.4 | N05BB01: Hydroxyzine | 1.2 | N03AG04: Vigabatrin | 1.6 |
|  | **6** | N03AA02: Phenobarbital | 1.1 | N03AA02: Phenobarbital | 1 | N03AA02: Phenobarbital | 1.3 |
|  | **7** | N05AA02: Levomepromazine | 0.5 | N05AA02: Levomepromazine | 0.4 | N03AF02: Oxcarbazepine | 0.5 |
|  | **8** | N03AF02: Oxcarbazepine | 0.4 | N05BA09: Clobazam | 0.4 | N05AA02: Levomepromazine | 0.5 |
|  | **9** | N05BA09: Clobazam | 0.4 | N03AF02: Oxcarbazepine | 0.3 | N03AE01: Clonazepam | 0.5 |
|  | **10** | N03AE01: Clonazepam | 0.4 | N03AE01: Clonazepam | 0.3 | N05BA09: Clobazam | 0.4 |
| **1-2 years** | **1** | N05BA01: Diazepam | 17.8 | N05BA01: Diazepam | 16.1 | N05BA01: Diazepam | 19.3 |
|  | **2** | N05BB01: Hydroxyzine | 11.1 | N05BB01: Hydroxyzine | 8.4 | N05BB01: Hydroxyzine | 13.7 |
|  | **3** | N03AG01: Valproic acid | 7.4 | N03AG01: Valproic acid | 6.9 | N03AG01: Valproic acid | 7.8 |
|  | **4** | N03AX14: Levetiracetam | 3.1 | N03AX14: Levetiracetam | 2.7 | N03AX14: Levetiracetam | 3.4 |
|  | **5** | N05BX92: Passiflora | 2.7 | N05BX92: Passiflora | 2.4 | N05BX92: Passiflora | 3.0 |
|  | **6** | N03AG04: Vigabatrin | 1.1 | N03AG04: Vigabatrin | 1 | N03AA02: Phenobarbital | 1.3 |
|  | **7** | N03AA02: Phenobarbital | 1.1 | N03AF02: Oxcarbazepine | 1 | N03AG04: Vigabatrin | 1.2 |
|  | **8** | N03AF02: Oxcarbazepine | 0.9 | N03AA02: Phenobarbital | 0.9 | N05AA02: Levomepromazine | 0.9 |
|  | **9** | N05BA09: Clobazam | 0.8 | N05BA09: Clobazam | 0.8 | N05BA09: Clobazam | 0.9 |
|  | **10** | N03AF01: Carbamazepine | 0.8 | N05BA12: Alprazolam | 0.7 | N03AF02: Oxcarbazepine | 0.8 |
| **3-5 years** | **1** | N05BB01: Hydroxyzine | 26.0 | N05BB01: Hydroxyzine | 19.6 | N05BB01: Hydroxyzine | 32.1 |
|  | **2** | N05BA01: Diazepam | 14.4 | N05BA01: Diazepam | 13.0 | N05BA01: Diazepam | 15.8 |
|  | **3** | N03AG01: Valproic acid | 9.6 | N03AG01: Valproic acid | 8.8 | N03AG01: Valproic acid | 10.4 |
|  | **4** | N05AX08: Risperidone | 4.3 | N05BX92: Passiflora | 3.7 | N05AX08: Risperidone | 6.3 |
|  | **5** | N06BA04: Methylphenidate | 4.0 | N03AX14: Levetiracetam | 3.4 | N06BA04: Methylphenidate | 5.8 |
|  | **6** | N05BX92: Passiflora | 4.0 | N06BA04: Methylphenidate | 2.2 | N05BX92: Passiflora | 4.3 |
|  | **7** | N03AX14: Levetiracetam | 3.7 | N05AX08: Risperidone | 2.1 | N03AX14: Levetiracetam | 3.9 |
|  | **8** | N03AF01: Carbamazepine | 1.5 | N03AF01: Carbamazepine | 1.5 | N05BA12: Alprazolam | 1.6 |
|  | **9** | N05BA12: Alprazolam | 1.5 | N03AF02: Oxcarbazepine | 1.4 | N03AF01: Carbamazepine | 1.6 |
|  | **10** | N03AF02: Oxcarbazepine | 1.5 | N05BA12: Alprazolam | 1.3 | N05BA05: Potassium clorazepate | 1.6 |
| **6-8 years** | **1** | N06BA04: Methylphenidate | 53.1 | N06BA04: Methylphenidate | 27.2 | N06BA04: Methylphenidate | 77.3 |
|  | **2** | N05BB01: Hydroxyzine | 29.0 | N05BB01: Hydroxyzine | 23.0 | N05BB01: Hydroxyzine | 34.7 |
|  | **3** | N05AX08: Risperidone | 17.3 | N03AG01: Valproic acid | 10.7 | N05AX08: Risperidone | 27.4 |
|  | **4** | N03AG01: Valproic acid | 11.7 | N05BA01: Diazepam | 10.1 | N03AG01: Valproic acid | 12.6 |
|  | **5** | N05BA01: Diazepam | 10.8 | N05AX08: Risperidone | 6.5 | N05BA01: Diazepam | 11.5 |
|  | **6** | N06BA09: Atomoxetine | 5.1 | N03AX14: Levetiracetam | 4.5 | N06BA09: Atomoxetine | 7.7 |
|  | **7** | N05BA05: Potassium clorazepate | 4.8 | N05BX92: Passiflora | 4.2 | N05AX12: Aripiprazole | 7.5 |
|  | **8** | N05AX12: Aripiprazole | 4.7 | N05BA05: Potassium clorazepate | 4.0 | N05BA05: Potassium clorazepate | 5.5 |
|  | **9** | N03AX14: Levetiracetam | 4.7 | N03AF01: Carbamazepine | 2.7 | N03AX14: Levetiracetam | 4.9 |
|  | **10** | N05BX92: Passiflora | 4.4 | N06BA09: Atomoxetine | 2.4 | N05BX92: Passiflora | 4.6 |
| **9-11 years** | **1** | N06BA04: Methylphenidate | 111.9 | N06BA04: Methylphenidate | 59.9 | N06BA04: Methylphenidate | 160.8 |
|  | **2** | N05AX08: Risperidone | 30.4 | N05BB01: Hydroxyzine | 23.9 | N05AX08: Risperidone | 47.9 |
|  | **3** | N05BB01: Hydroxyzine | 27.4 | N05BA01: Diazepam | 14.1 | N05BB01: Hydroxyzine | 30.6 |
|  | **4** | N06BA09: Atomoxetine | 15.2 | N05AX08: Risperidone | 11.8 | N06BA09: Atomoxetine | 22.8 |
|  | **5** | N05BA01: Diazepam | 14.1 | N03AG01: Valproic acid | 10.7 | N05AX12: Aripiprazole | 15.6 |
|  | **6** | N03AG01: Valproic acid | 12.6 | N05BA05: Potassium clorazepate | 9.5 | N03AG01: Valproic acid | 14.4 |
|  | **7** | N05BA05: Potassium clorazepate | 10.4 | N06BA09: Atomoxetine | 7.2 | N05BA01: Diazepam | 14.1 |
|  | **8** | N05AX12: Aripiprazole | 10.0 | N05BX92: Passiflora | 5.7 | N05BA05: Potassium clorazepate | 11.3 |
|  | **9** | N06AB03: Fluoxetine | 7.2 | N06AB03: Fluoxetine | 5.4 | N06BA12: Lisdexamfetamine | 9.5 |
|  | **10** | N06AB06: Sertraline | 6.5 | N06AB06: Sertraline | 4.9 | N06AB03: Fluoxetine | 8.9 |
| **12-14 years** | **1** | N06BA04: Methylphenidate | 142.4 | N06BA04: Methylphenidate | 73.2 | N06BA04: Methylphenidate | 207.4 |
|  | **2** | N05AX08: Risperidone | 39.0 | N05BA01: Diazepam | 34 | N05AX08: Risperidone | 57.8 |
|  | **3** | N05BA01: Diazepam | 29.0 | N06AB03: Fluoxetine | 21.9 | N06BA09: Atomoxetine | 29.5 |
|  | **4** | N06AB03: Fluoxetine | 20.5 | N05AX08: Risperidone | 19.0 | N05BA01: Diazepam | 24.3 |
|  | **5** | N06BA09: Atomoxetine | 19.5 | N05BB01: Hydroxyzine | 18.9 | N05AX12: Aripiprazole | 22.5 |
|  | **6** | N05BB01: Hydroxyzine | 18.9 | N06AB06: Sertraline | 16.3 | N06AB03: Fluoxetine | 19.1 |
|  | **7** | N06AB06: Sertraline | 17.3 | N05BA05: Potassium clorazepate | 14.5 | N06BA12: Lisdexamfetamine | 18.9 |
|  | **8** | N05AX12: Aripiprazole | 16.6 | N03AG01: Valproic acid | 12 | N05BB01: Hydroxyzine | 18.8 |
|  | **9** | N03AG01: Valproic acid | 14.2 | N05AX12: Aripiprazole | 10.3 | N06AB06: Sertraline | 18.3 |
|  | **10** | N05BA05: Potassium clorazepate | 14.2 | N05BA06: Lorazepam | 9.1 | N03AG01: Valproic acid | 16.3 |
| **15-17 years** | **1** | N06BA04: Methylphenidate | 119.8 | N05BA01: Diazepam | 98.3 | N06BA04: Methylphenidate | 172.5 |
|  | **2** | N05BA01: Diazepam | 73.8 | N06BA04: Methylphenidate | 63.2 | N05AX08: Risperidone | 51.9 |
|  | **3** | N06AB03: Fluoxetine | 39.4 | N06AB03: Fluoxetine | 52.3 | N05BA01: Diazepam | 51.1 |
|  | **4** | N05AX08: Risperidone | 37.0 | N06AB06: Sertraline | 35.4 | N06AB03: Fluoxetine | 27.3 |
|  | **5** | N06AB06: Sertraline | 29.6 | N05BA12: Alprazolam | 33.1 | N06AB06: Sertraline | 24.3 |
|  | **6** | N05BA12: Alprazolam | 23.8 | N05BA06: Lorazepam | 29.5 | N05AX12: Aripiprazole | 23.7 |
|  | **7** | N05BA06: Lorazepam | 22.1 | N05AX08: Risperidone | 21.1 | N06BA09: Atomoxetine | 22.4 |
|  | **8** | N05AX12: Aripiprazole | 19.7 | N03AX11: Topiramate | 18.2 | N03AG01: Valproic acid | 18.4 |
|  | **9** | N05AH04: Quetiapine | 16.8 | N05AH04: Quetiapine | 17.5 | N05AH04: Quetiapine | 16.3 |
|  | **10** | N03AG01: Valproic acid | 16.4 | N06AA09: Amitriptyline | 16.7 | N06BA12: Lisdexamfetamine | 15.9 |
| **0-17 years** | **1** | N06BA04: Methylphenidate | 41.0 | N05BA01: Diazepam | 26.0 | N06BA04: Methylphenidate | 58.7 |
|  | **2** | N05BA01: Diazepam | 22.2 | N06BA04: Methylphenidate | 22.2 | N05AX08: Risperidone | 20.8 |
|  | **3** | N05BB01: Hydroxyzine | 17.7 | N05BB01: Hydroxyzine | 15.3 | N05BB01: Hydroxyzine | 19.9 |
|  | **4** | N05AX08: Risperidone | 14.2 | N06AB03: Fluoxetine | 10.8 | N05BA01: Diazepam | 18.7 |
|  | **5** | N06AB03: Fluoxetine | 9.2 | N06AB06: Sertraline | 7.7 | N06BA09: Atomoxetine | 9.7 |
|  | **6** | N03AG01: Valproic acid | 7.5 | N05AX08: Risperidone | 7.2 | N03AG01: Valproic acid | 8.3 |
|  | **7** | N06AB06: Sertraline | 7.3 | N05BA05: Potassium clorazepate | 6.8 | N05AX12: Aripiprazole | 7.9 |
|  | **8** | N05BA05: Potassium clorazepate | 6.6 | N05BA12: Alprazolam | 6.7 | N06AB03: Fluoxetine | 7.8 |
|  | **9** | N06BA09: Atomoxetine | 6.6 | N03AG01: Valproic acid | 6.6 | N06AB06: Sertraline | 6.9 |
|  | **10** | N05AX12: Aripiprazole | 5.9 | N05BA06: Lorazepam | 6.3 | N05BA05: Potassium clorazepate | 6.4 |
